# A Review on Traditional Processes and Laser Powder Bed Fusion of Aluminum Alloy Microstructures, Mechanical Properties, Costs, and Applications

**DOI:** 10.3390/ma17112553

**Published:** 2024-05-25

**Authors:** Xin Wang, Dongyun Zhang, Ang Li, Denghao Yi, Tianci Li

**Affiliations:** 1 Institute of Laser Engineering, School of Physics and Optoelectronic Engineering, Beijing University of Technology, Beijing 100124, China; wx369806199@hotmail.com (X.W.); yidh@emails.bjut.edu.cn (D.Y.); tianci.li@bjut.edu.cn (T.L.); 2 Beijing Engineering Research Center of 3D Printing for Digital Medical Health, Beijing 100124, China; 3 China United Gas Turbine Technology Co., Ltd., Beijing 100016, China; 15210603161@163.com; 4 School of Materials Science and Engineering, Tsinghua University, Beijing 100084, China

**Keywords:** aluminum, LPBF, additive manufacturing, mechanical property, cost

## Abstract

Due to its lightweight, high strength, good machinability, and low cost, aluminum alloy has been widely used in fields such as aerospace, automotive, electronics, and construction. Traditional manufacturing processes for aluminum alloys often suffer from low material utilization, complex procedures, and long manufacturing cycles. Therefore, more and more scholars are turning their attention to the laser powder bed fusion (LPBF) process for aluminum alloys, which has the advantages of high material utilization, good formability for complex structures, and short manufacturing cycles. However, the widespread promotion and application of LPBF aluminum alloys still face challenges. The excellent printable ability, favorable mechanical performance, and low manufacturing cost are the main factors affecting the applicability of the LPBF process for aluminum alloys. This paper reviews the research status of traditional aluminum alloy processing and LPBF aluminum alloy and makes a comparison from various aspects such as microstructures, mechanical properties, application scenarios, and manufacturing costs. At present, the LPBF manufacturing cost for aluminum alloys is 2–120 times higher than that of traditional manufacturing methods, with the discrepancy depending on the complexity of the part. Therefore, it is necessary to promote the further development and application of aluminum alloy 3D printing technology from three aspects: the development of aluminum matrix composite materials reinforced with nanoceramic particles, the development of micro-alloyed aluminum alloy powders specially designed for LPBF, and the development of new technologies and equipment to reduce the manufacturing cost of LPBF aluminum alloy.

## 1. Introduction

As global concern for carbon emissions continues to increase, green and low-carbon development has become an important strategy. Aluminum alloy, a lightweight material with high mechanical properties, favorable processing performance, a large reserve, and low price [1,2,3], has gradually replaced traditional steel materials and is widely used in aerospace, automotive, construction, and other fields [4,5,6,7,8,9]. For example, 7075 and 2024 aluminum alloys are commonly used in the manufacture of skins, structural components, and fasteners in the aerospace industry, and 5052 and 5038 aluminum alloys are commonly used in the manufacture of ships, offshore platforms, and vehicles. In addition, Al-based composites are widely used in the manufacture of electronic packaging, thermal control components, and ventral fin of fighters.

**Figure 1:**
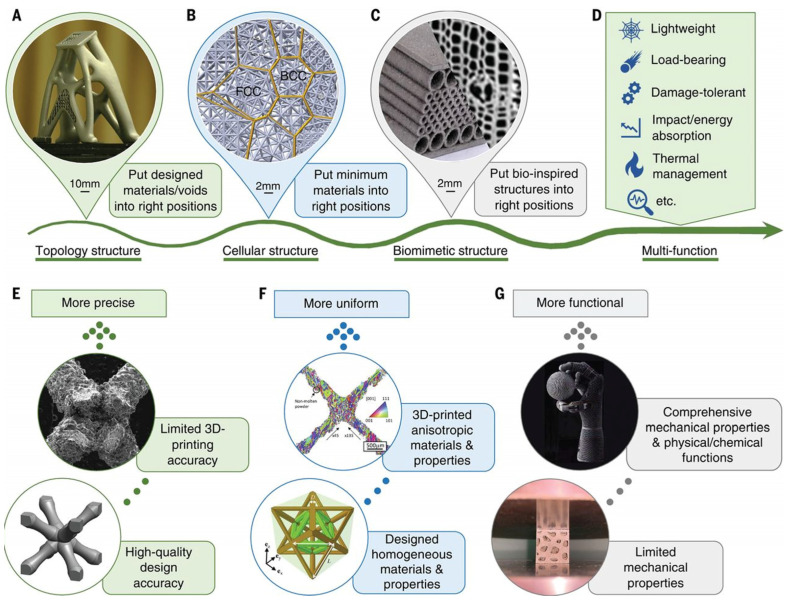
Multifunctional LPBF additive manufacturing through structural design and innovation to reflect the idea of “unique structures printed for unique functions” [20]. (**A**) A topology-optimized satellite bracket filled with lattices, (**B**) a crystal-inspired cellular structure containing hierarchical face-centered cubic (FCC) and (**C**) body-centered cubic (BCC) lattices, and (**D**) a functional graded sandwich structure inspired by the Norway spruce stem. Three categories of existing gaps and development directions in multifunctional laser-metal AM, in terms of (**E**) manufacturing accuracy, (**F**) material and property anisotropy, and (**G**) diversity of functions.

However, traditional manufacturing processes for aluminum alloys suffer from problems such as low raw material utilization, complex procedures, and long processing cycles. These issues are particularly pronounced for complex-shaped and high-quality formed parts. Therefore, additive manufacturing technology, which can achieve near-net shape or even direct forming, has gradually become one of the important methods for aluminum alloy manufacturing [10,11,12,13]. Among them, the laser powder bed fusion (LPBF) process has developed rapidly and has the advantages of high material utilization [14,15], complex structure formation [16,17,18], and short preparation cycles [19]. It has made significant breakthroughs in the manufacture of materials such as titanium alloys and superalloys (Figure 1) [20,21,22].

The number of available aluminum alloy grades for LPBF use is gradually increasing, from the initial AlSi alloy to the current 2XXX and 7XXX aluminum alloys, and even specific aluminum alloys developed for LPBF [23,24]. Weldability is a crucial factor that affects the performance of aluminum alloys made by LPBF. Aluminum alloys with good weldability also tend to exhibit better LPBF performance. Aluminum alloys are classified into various types, including AlSi alloys (4XXX), which are known for their good weldability. This is due to the high Si content (approximately 7–13%) in the alloy. The presence of Si reduces the coefficient of thermal expansion and contraction, improves flowability, and lowers the tendency towards thermal cracking. On the other hand, 2XXX aluminum alloy, which contains a high amount of copper (with a high melting point), has a wider two-phase region and is more susceptible to hot cracking. The 7XXX aluminum alloy has a complex composition, with elements such as Zn, Mg, and Cu that enhance its mechanical properties but also increase its susceptibility to cracking, making it more difficult to weld.

Furthermore, LPBF aluminum alloys have undergone significant development in their mechanical properties, increasing from 200 MPa to 600 MPa [25,26]. Despite these advancements, there remain challenges in the application of LPBF aluminum alloys. Specifically, in the aerospace industry, aluminum alloys are primarily utilized for load-bearing structures [27,28] and must exhibit high tensile, compression, and fatigue mechanical properties. Currently, the majority of aluminum alloy parts produced by LPBF do not meet mechanical property requirements [29,30]. Additionally, in industries such as automotive, electronics, and construction, manufacturing costs are a primary concern for companies. While traditional processing technology for aluminum alloys is relatively mature and has lower cost control, the aluminum alloy LPBF process has a shorter development time but higher manufacturing costs. The development and application of the aluminum alloy LPBF process are mainly restricted by its mechanical properties and manufacturing costs.

This paper reviews the research status of traditional aluminum alloy processing and LPBF aluminum alloy. It compares them based on microstructures, mechanical properties, application scenarios, and manufacturing costs. This paper summarizes the development opportunities of LPBF aluminum alloy for the future. It provides an important reference for promoting further development and application of LPBF aluminum alloy.

## 2. Research Status of Traditional Manufacturing Processes for Aluminum Alloy

### 2.1. Traditional Aluminum Alloy Grades

Aluminum alloy, as a lightweight and high-strength material, has been widely used in aviation, automotive, construction, and electronics. According to processing methods, it can be divided into deformation aluminum alloys and casting aluminum alloys. According to the chemical element composition, deformation aluminum alloys can be further divided into 1XXX to 8XXX (in accordance with the national standard GB/T3190-2020 [31]), with 2XXX series, 5XXX series, 6XXX series, and 7XXX aluminum alloys being the most commonly used.

The 2XXX aluminum alloy [32,33,34] is an aluminum–copper alloy, with copper, magnesium, manganese, and other elements as its main components. Its high hardness, large strength, good toughness, good thermal conductivity, and corrosion resistance make it an ideal material for manufacturing structural parts and high-strength products in aviation, automotive, railway, ships, construction, and other fields. The bodies of Boeing 787 and Airbus A380, the body and chassis of Tesla electric cars, as well as building wall and roof panels, window frames, doors, electronic device casings, and heat sinks are often made of 2XXX aluminum alloy. The 5XXX aluminum alloy [35,36,37] is an aluminum–magnesium alloy, with magnesium, manganese, and chromium as its main components, possessing moderate strength, high elongation, good corrosion resistance, and weldability. This material is widely used in shipbuilding, automotive, aviation, construction, packaging, and other fields, such as the body of Boeing 747 and Airbus A320, engine covers and doors of BMW and Audi cars, etc. The 6XXX aluminum alloy [38,39,40] is an aluminum–magnesium–silicon alloy, with magnesium, silicon, manganese, and other elements as its main components. It combines the advantages of 4XXX and 5XXX aluminum alloys, with high strength, good corrosion resistance, and ductility, and is widely used in automotive, aviation, construction, sports equipment, and other fields. The body of Boeing 777 and Airbus A350, the body and chassis of Porsche 911, and other products are made of 6XXX aluminum alloy. The 7XXX aluminum alloy [41,42,43] is an aluminum–zinc alloy, with zinc, magnesium, copper, and other elements as its main components, with advantages such as high hardness, high strength, and good corrosion resistance, because of which it is widely used in the manufacturing of aircraft structural components and pressure equipment. For example, the upper and lower wing panels, ribs, and long spars are all made of 7XXX aluminum alloy.

Casting aluminum alloys include aluminum–silicon casting alloys [44,45], aluminum–magnesium alloys [46], aluminum–zinc alloys, and aluminum–manganese alloys [47]. Among them, A360, A380, and A390 aluminum–silicon casting alloys have good processing performance, and they exhibit excellent mechanical properties in castings, suitable for the manufacture of complex casting structures. ADC12 aluminum–silicon casting alloy has good mechanical properties, especially wear resistance and corrosion resistance, and is widely used in automobile engines, electrical enclosures, and other fields. AZ91D aluminum–magnesium alloy has excellent strength and toughness and is commonly used in automotive parts, aerospace, and other fields.

### 2.2. Traditional Processes, Mechanical Properties, and Applications

Traditional aluminum alloy manufacturing processes include die casting, forging, and rolling, which are applicable to different grades of aluminum alloy. As a result, there are significant differences in the type, material properties, and application scenarios of aluminum alloy components produced by these methods.

Die casting is an important manufacturing process for aluminum alloy, especially for casting aluminum alloy. For example, Tesla has recently proposed an integrated die casting technology that utilizes the excellent properties of aluminum alloys, such as high strength and high elongation, to achieve high-pressure die casting under a vacuum. This technology has broad prospects for the production of large-size aluminum alloy parts. Integrated die casting often requires aluminum alloys with high strength and high elongation. For instance, researchers have developed AlSiCuMgMn alloy, which, after being subjected to 510 °C solution treatment for 30 min and 170 °C aging for 12 h, has a yield strength and ultimate tensile strength of 321 MPa and 425 MPa, respectively, and an elongation of 11.3%, which is 150% higher than that of the existing die casting aluminum alloys [48]. Also, the development of heat-treatment-free aluminum alloys is one of the important directions of integrated die casting technology. Comparing the tensile properties of AlSiCuMgMn alloy before and after heat treatment, it was found that the tensile strength of the alloy only increased from 310 MPa to 375 MPa after heat treatment, and the elongation decreased from 6.9% to 4.3% [49].

Forging is commonly used in the production of high-performance aluminum alloy bearing structures, such as automobile wheel hubs, connecting rods, control arms, and aircraft landing gear, mainly using deformation aluminum alloys. Advanced forging techniques such as precision forging, closed-die forging, and isothermal forging have been developed. Among them, precision forging can achieve the near-net shape of parts, with a material utilization rate increased by 60%, and higher production efficiency. Compared to the casting process, the forging process can effectively improve the mechanical properties of aluminum alloys. After undergoing the forging process, ADC12 aluminum alloy has a grain size of 26.54 mm. Its tensile strength and elongation can research 287.75 MPa and 4.4%, which are 19.8% and 340% higher than those of casting aluminum alloys, respectively [50]. After precision die forging, the tensile strength of 7A04 aluminum alloy exceeds 560 MPa, and the elongation rate is over 6%.

Rolling is a primary way to prepare aluminum foils and sheets, mainly using deformation aluminum alloys. Rolling is further divided into cold-rolling and hot-rolling processes, and different combinations of these processes can be used for different processing requirements. For example, 6A16 aluminum alloy typically needs to undergo multiple procedures including casting, hot-rolling, intermediate annealing, cold-rolling, solution treatment, and aging to obtain a sheet with a grain size of about 23 μm, a tensile strength of 160–261 MPa, and an elongation of 15–31.5% [51].

Aluminum alloy components made by traditional processing methods are widely used in fields such as aerospace, automotive, electronics, and construction. For example, aluminum alloys account for as much as 81% of the Boeing 737 aircraft, used to manufacture various types of aircraft skins, landing gears, and fuel tanks. In the automotive field, aluminum alloy can be used to manufacture internal cylinders of engines, external covers, brake noise shields, and body frameworks, effectively achieving the lightweight of the vehicle body. Moreover, replacing traditional steel plates with aluminum alloys can significantly reduce the weight of the car body by up to 50% maximum. In the electronics industry, aluminum alloys are commonly used to manufacture various types of wires, electronic device casings, and other components. Additionally, aluminum alloys are an important building material, which not only has good structural strength but also has certain esthetics, making it easy to color and weld.

### 2.3. Cost of Traditional Aluminum Alloy Manufacturing Processes

The cost control of aluminum alloy parts manufactured by traditional processes is relatively strict, and prices mainly fluctuate with the raw material prices. According to data from the website of the Ministry of Industry and Information Technology of China, the unit price of pure aluminum per kilogram was 18.62 CNY (2.57 USD) in the first three quarters of 2023 [52,53]. Different grades of aluminum alloy have different smelting difficulties and costs, and the raw material prices also vary greatly. Table 1 shows the prices of various grades of aluminum alloys. The price of 7055 alloys is much higher than that of other aluminum alloys, mainly because of its difficulty in casting and limited application scenarios, which makes it difficult to control manufacturing costs. In addition, factors such as impurity content, type of other elements, and raw material status will also affect the price of aluminum alloy raw materials.

In addition to raw material costs, the subsequent secondary processing process is also an important factor affecting the production cost of aluminum alloys. For example, typical secondary processing methods such as integrated die casting require large die casting equipment and sand molds to be used together, and the investment in large die casting equipment will result in higher one-time costs. With the increase in production volume and technological optimization, the manufacturing cost of aluminum alloys will gradually decrease.

## 3. Research Status of LPBF Process for Aluminum Alloy

The LPBF process [54,55,56,57] is characterized by high laser energy density and high scanning speed, resulting in both heat conduction welding and deep penetration welding during laser–powder interaction. The high-speed movement of the small laser spot creates a large temperature gradient within the melt pool, where the instantaneous temperature is much higher than the melting point of the material. After the laser exits, rapid cooling occurs at a rate of 10^4^–10^6^ K/s. This rapid heating and cooling process results in significant residual stresses within the material, making it difficult to use LPBF for Al alloys with high crack sensitivity and cold cracking susceptibility. In addition, aluminum alloys that require the addition of welding wire for welding are also difficult to apply to the LPBF process. There are a limited number of traditional aluminum alloy grades that can meet the above requirements, and they often have low strength and high ductility. Heat treatment is therefore essential. On the one hand, heat treatment can improve the mechanical properties of aluminum alloys. On the other hand, it can relieve the internal stresses generated during the LPBF process, thereby improving the fatigue performance of the components and ultimately meeting the service conditions.

**Figure 2:**
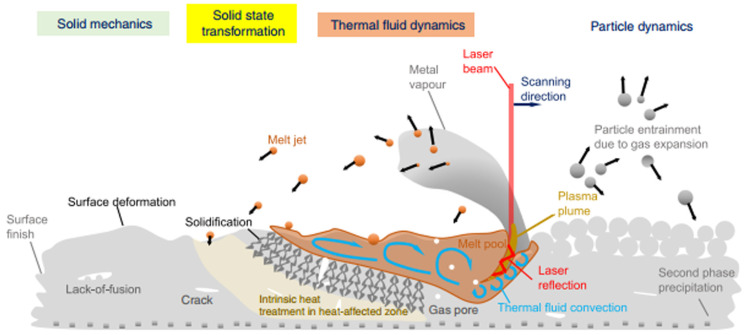
A schematic illustration of multi-scale, multi-physics phenomena in LPBF [57].

The schematic illustration of LPBF is shown in Figure 2. A molten pool is formed by rapid interaction between laser and powder, and the laser spot moves quickly and prints layer by layer according to a predetermined path. The new powder is then laid on the surface of the printed layer using a scraper, and this process is repeated until the desired shape is achieved [57]. The LPBF process can be used to print parts with small features and high surface precision, and the remaining powder can be recycled for printing [58,59,60], achieving high material utilization. As the size of the molded part is limited by the size of the powder bed and the accuracy of the powder spreading equipment, the larger the size of the molded part, the greater the amount of powder required, and the harder it is to ensure the evenness of the powder spreading. Therefore, this process is generally used to manufacture parts with dimensions below the “meter level” [61].

### 3.1. Defects and Microstructures

Aluminum alloy possesses several distinct features, including low density, low laser absorption rate (approximately 9% for CO_2_ laser), and high thermal conductivity (reaching up to 237 W·m^−1^·K^−1^, which is 16 times that of titanium). During the LPBF process of aluminum alloy, the laser experiences intense interactions between powder particles, leading to complex physical and chemical phenomena such as laser energy absorption and scattering, heat and force conduction, phase transformation, and Marangoni convection [62,63,64]. These phenomena bring about metallurgical defects such as balling [65,66], residual stress [67], alloy element evaporation [30], pores, and cracks [68,69]. As a result, LPBF technology faces significant challenges in preparing high-performance aluminum alloy components, making it a classic case of difficult-to-print material [62,70]. Furthermore, aluminum has a high affinity for oxygen, which makes it easy to form an oxide film on the surface of the melt and exacerbate the occurrence of defects. A substantial body of research has been conducted to investigate the control of porosity in LPBF aluminum alloys. This research has employed a range of methods, including the increase in laser power, the optimization of scanning strategies, the adjustment of alloy composition, and the utilization of hot isostatic pressing treatments. These studies have been extensively reviewed and will not be further discussed in this paper [30,56,71].

**Figure 3:**
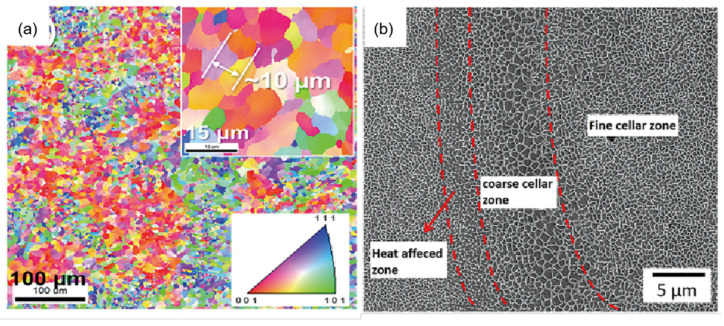
(**a**) Inverse polar figure obtained by EBSD; (**b**) LPBFed AlSi alloy microstructure at high magnification [78].

Among aluminum alloys, AlSi alloys demonstrate better LPBF formability due to their good casting and welding properties [72]. The main factors affecting LPBF aluminum alloy formability are laser energy density [73], scanning and printing strategies [74,75], and heat treatment processes [76,77]. When the laser energy density increases from 150 J/m to 200 J/m, the density of the LPBF-printed AlSi7Mg alloy component rises from 94.6% to 99.6% [72]. However, further increasing the laser energy density will lead to increased evaporation of low melting point elements such as aluminum and magnesium, making it difficult for bubbles to escape and causing a decrease in component density. In the LPBF process, AlSi alloys demonstrate similar microstructure evolution rules, i.e., forming cellular structures enriched with aluminum. The high-melting-point silicon particles are the first to nucleate unevenly in the molten pool with increasing temperature, followed by nucleation and growth of α-Al in the area around the silicon particles. The continuous solidification of α-Al leads to an increased concentration of silicon in the residual liquid phase, causing the liquid composition to gradually move toward the eutectic range and form an AlSi eutectic structure [72]. Figure 3b shows the microstructure of the AlSi alloy prepared by LPBF, with the diameter of the small cellular structure being approximately 500 nm [78]. Moreover, the grain size of the alloy, as shown in Figure 3a, is approximately 10 μm, which is much smaller than that of the cast alloy [79]. In addition, traditional Al-Mg-Si alloys belong to the aging strengthening type alloys, and their precipitation strengthening sequence is α (Al) → GP area → β’’ → β’ → β [80]. Among them, the GP region is composed of Mg and Si clusters, which are completely coherent with the FCC Al matrix. The β’’ and β’ phases are metastable Mg-Si with different stoichiometry, while the β phase is the stable Mg2Si phase. After T6 heat treatment, the fine cellular structure is coarsened as a whole in LPBF-fabricated AlSi10Mg alloy, and the eutectic Si network spheroidizes into irregularly shaped Si particles (200 nm–4 μm). As the aging treatment progresses, the needle-like β’’ phase precipitates from the AlSi10Mg matrix, the length of β’’ phase is mostly <10 nm, and the GP zones are around 2–3 nm in size after age treatment (160 °C for 10 h) [81]. But the average size of β’’ phase is relatively large, with 20 ± 2.9 nm after solid solution and peak aging treatment in the cast A357 alloy [82].

**Figure 4:**
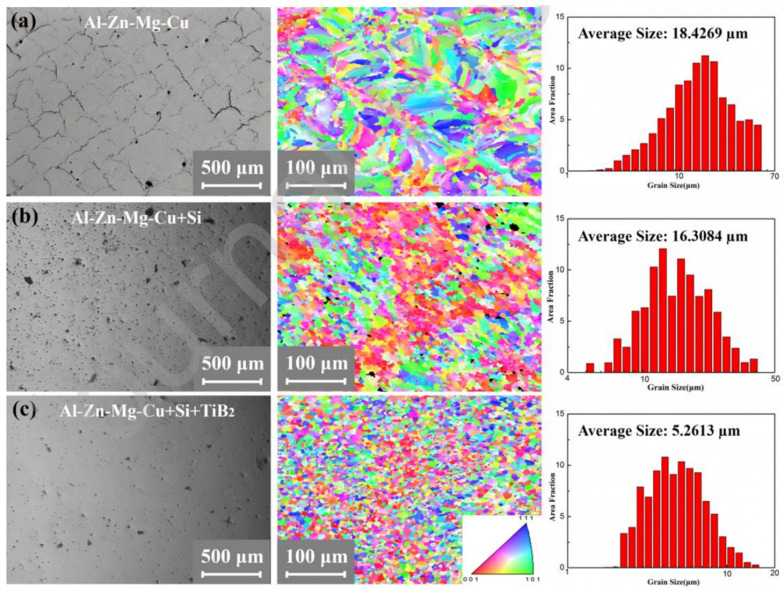
OM images, EBSD images, and grain size distributions of [90]: (**a**) the as-printed Al-Zn-Mg-Cu alloy; (**b**) the Al-Zn-Mg-Cu + Si alloy, and (**c**) the Al-Zn-Mg-Cu + Si + TiB2 alloy.

As AlSi alloys cast aluminum alloys, their mechanical properties are lower and cannot meet the high-performance requirements of the aerospace field. Therefore, some scholars have begun to study precipitation-strengthened aluminum alloys such as AlZn [83,84], AlCu [85,86], and AlMn [23,87] systems prepared by LPBF technology. However, these aluminum alloys are prone to cracking and porosity during LPBF forming [84], especially 7XXX aluminum alloys, which exhibit higher sensitivity to cracking and poorer formability [84,88,89]. In order to overcome these difficulties, Martin et al. proposed a method of modifying the aluminum alloy powder surface with added nanoparticles, which effectively improves the microstructure of the aluminum alloy and obtains high-quality, non-cracking, high-strength aluminum alloy components [88]. By electrostatically assembling zirconium-based nano-nucleating agents uniformly on the surface of 7075 aluminum alloy powder, the Zr reacts with Al to form in situ Al3Zr during the printing process. This provides an ideal low-energy potential barrier for heterogeneous nucleation sites, promoting the transformation of columnar crystals into fine equiaxed crystals, reducing the impact of solidification thermal contraction, and obtaining non-cracking, high-strength aluminum alloy components [88]. In addition, adding submicron Si and TiB2 particles can also reduce internal defects in aluminum alloys. Si elements reduce the solidification temperature range of AlZnMgCu alloys, while TiB2 particles provide ideal nucleation sites, resulting in fine grains and improved mechanical properties of aluminum alloys (Figure 4) [90].

In addition, some scholars have designed high-performance aluminum alloy materials suitable for LPBF technology. Among them, AlMgScZr alloys reinforced by rare earth element scandium showed great potential during the preparation process [24]. This is because Al3 (Zr, Sc) nanoparticles formed during the printing process will segregate at the grain boundaries, increasing the heterogeneity of the alloy’s microstructure [91].

### 3.2. Mechanical Properties

**Figure 5:**
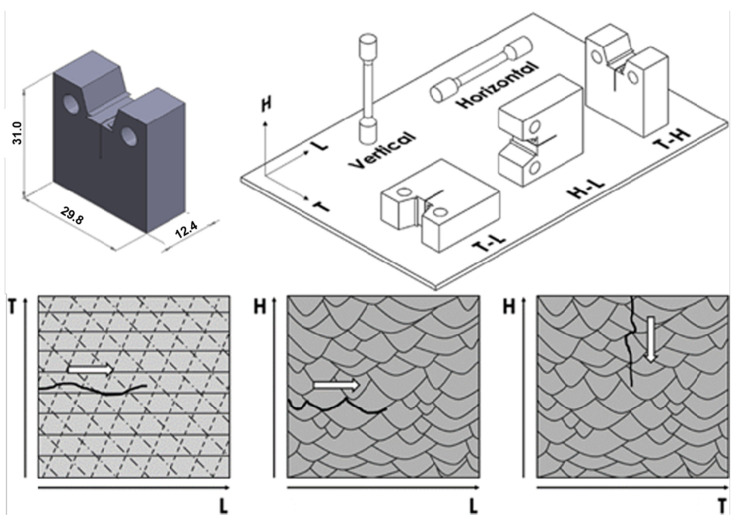
Crack propagation path of A347 alloy printed by LPBF process [94].

The laser energy density is a key factor that affects the tensile properties of aluminum alloys. Increasing the laser energy density can intensify Marangoni convection in the molten pool and promote the uniform distribution of Si particles. Studies have shown that the AlSi7Mg alloy printed with a laser energy density of 200 J/m has a tensile strength of 475.8 MPa and an elongation rate of 6.4% [92]. In addition, scanning and printing strategies also have an impact on the mechanical properties of aluminum alloys. Using an island scanning strategy can improve the tensile strength and elongation rate of aluminum alloys [93]. When there is an angle between the island and inter-layer scanning directions, the impact of direct scanning and island scanning strategies on the tensile properties of aluminum alloys can be ignored [79]. Printing in the horizontal or vertical direction has a small impact on the tensile strength of A357 alloy, but a significant impact on its fracture toughness. For example, when the crack propagates along the bottom edge of the molten pool, the fracture toughness of the alloy is the lowest (4.1 kJ/m^2^), which is only one-third of the value when the crack passes through the molten pool (Figure 5) [94].

Furthermore, aluminum alloys printed by LPBF technology need to undergo heat treatment [95], as the high-temperature gradient and rapid cooling during the LPBF printing process can cause excessive residual stress inside the material, which can cause a brittle fracture of the material. Therefore, some scholars have explored the heat treatment process of LPBF aluminum alloys. For example, after heat treatment at 300 °C for 6 h, the AlSi12 alloy changed from brittle fracture to ductile fracture. At this time, the tensile strength and yield strength decreased by 43% and 29.8%, respectively, while the elongation rate increased by 20.5% [79]. After 160 °C/4 h aging treatment, the tensile strength of LPBF-printed A357 alloy reached 411 MPa, but the elongation rate decreased to 4.8% [94]. Increasing the heat treatment temperature can effectively reduce residual stress, improve the crystallinity, and reduce the differences between the surface and internal microstructures of aluminum alloys caused by different cooling rates [96]. However, when the heat treatment temperature exceeds the eutectic temperature of the alloy, the porosity will increase sharply [96]. In addition, research has shown that the corrosion resistance of AlSi10Mg alloy decreases with the increase in heat treatment temperature [97]. The as-built AlSi10Mg alloy has excellent corrosion resistance because the continuous eutectic Si network can protect the aluminum matrix. When the heat treatment temperature rises to 300 °C or above, the eutectic Si network fractures and spheroidizes into Si particles. During the corrosion process, Si particles and Al form micro batteries, accelerating the pitting effect of AlSi10Mg alloy. However, due to the uniformity of Si particle structure, local corrosion and selective infiltration erosion disappear at the melt pool boundary. This result can be obtained in Harrison’s solution [98], in 1 M HNO3 solution [99], and in 0.51 M NaCl [100].

**Figure 6:**
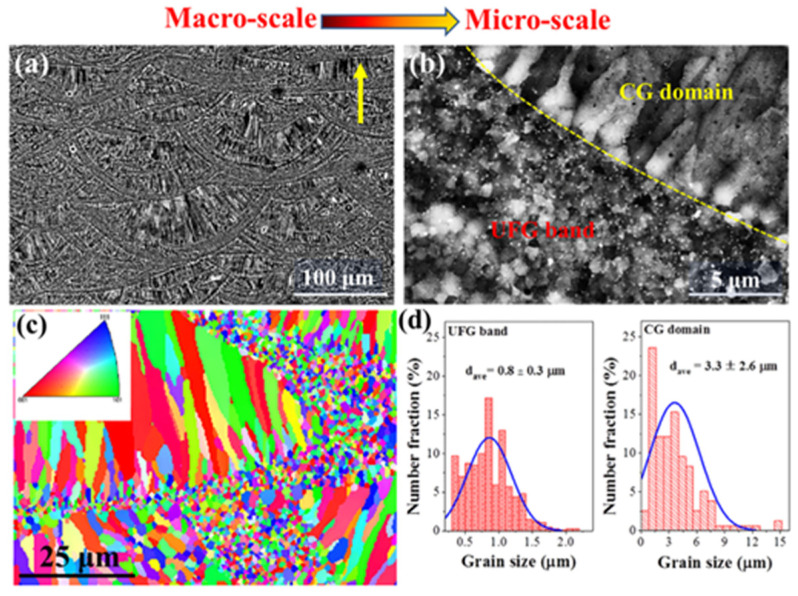
Microstructure of AlMgScZr alloy [101]. (**a**,**b**) SEM images showing the heterogeneous microstructure in the SLM-processed AlMgScZr alloy; (**c**) EBSD maps showing the heterogeneous α-Al grain structure; (**d**) grain size distributions in the UFG bands and CG domains.

As shown in Figure 6, the addition of nanoparticles can extend the crack propagation path and become an external factor to improve the strength and toughness of aluminum. The ultrafine grains produced at high cooling rates are an internal factor that improves the strength and toughness. Under the combined action of external and internal factors, the strength and fracture toughness of AlMgScZr alloy is significantly improved, comparable to that of 7075-T651 alloy [101]. In addition, some scholars have replaced Sc with Er elements, which have similar performance and lower cost, in the LPBF process for AlSi and AlMg alloys, and then prepared AlMgErZr alloys. After artificial aging, the elongation rate reached 15%, and the tensile strength exceeded 500 MPa [102,103]. This paper summarizes some related research, and Table 2 lists some of the existing data on the tensile properties of aluminum alloys prepared by LPBF technology. Moreover, after peak aging, the LPBF-fabricated AlMgErZr alloy exhibits good resistance to intergranular corrosion. The weight loss of peak aging Al4.5MgErZr alloy is only 6 mg/cm^2^ after accelerated sensitization (annealing at 100 °C for 7 days).

**Figure 7:**
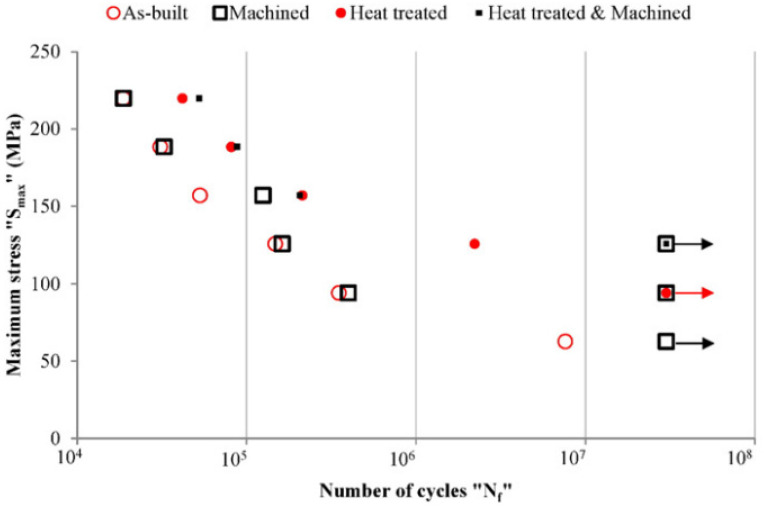
S-N curves of LPBF AlSi10Mg superimposed for all the investigated conditions [109].

In addition, some scholars have also studied the fatigue properties of aluminum alloys prepared by LPBF technology [104,105]. Studies have shown that the fatigue properties of the AlSi10Mg alloy prepared by LPBF technology are similar to those of the casting A360 alloy [106], and its fatigue strength can reach 120 MPa [107]. The key factors affecting the fatigue properties of LPBF aluminum alloys include porosity, microstructure, residual stress, and surface roughness. By adopting suitable process parameters, the density of AlSi10Mg alloy can reach 99.8% [106]. By the hot isostatic pressing process, almost completely dense aluminum-alloy-formed parts can be produced [108]. Some scholars have also explored the impact of heat treatment and surface processing on the fatigue properties of AlSi10Mg, as shown in Figure 7 [109]. The study found that although surface processing can improve the high-cycle fatigue properties of aluminum alloys, it has little effect on their low-cycle fatigue properties. Heat treatment can significantly improve the fatigue properties of aluminum alloys, which is mainly due to the improvement of elongation rate, microstructure transformation, and reduction in residual stress. In addition, the orientation angle of the formed parts also has an impact on their fatigue properties. When the angle is 0°, the fatigue performance of the part is the best [110,111], and this difference can be eliminated by heat treatment and bottom plate heating [106].

**Table 2:** Tensile properties of aluminum alloys fabricated by 3D printing.

Materials	Process	Heat Treatment	Modulus GPa	UTS MPa	Strain %	Ref.
AlSi10Mg	LPBF	As-fabricated	68 ± 3	396 ± 8	3.47 ± 0.6	[112]
		T6	66 ± 5	399 ± 7	/	
		As-fabricated	70.2 ± 1.2	267	9.1 ± 0.5	[26]
		As-fabricated	/	367.7	4	[113]
		As-fabricated	/	473	7.5	[114]
		160 °C/4 h	/	493	8.6	[115]
		540 °C/1 h +160 °C/4 h	/	323	15.3	
		450 °C/2 h	/	282.4 ± 6.1	13.4 ± 0.5	[116]
AlSi7Mg	LPBF	As-fabricated	/	475.8	6.4	[92]
AlSi12	LPBF	As-fabricated	/	418.9 ± 9.3	3.91 ± 0.3	[117]
		240 °C	/	369.3 ± 3.4	4.38 ± 0.16	
		As-fabricated	/	425.1 ± 24.7	12.1 ± 2.2	[118]
		2 h solution	/	190	25	[119]
		As-fabricated	/	325 ± 20	4.4 ± 0.7	[79]
		300 °C/6 h	/	228 ± 13	5.3 ± 0.7	
A357	LPBF	As-fabricated	/	398 ± 13	7.6 ± 1.8	[94]
		160 °C/4 h	/	411 ± 10	4.8 ± 0.8	
7075 + Zr	LPBF	T6	63~66	383~417	3.8~5.4	[88]
AlZn_5.43_Mg_2.65_Cu_1.4_ + Si + TiB_2_	LPBF	T6	/	566 ± 12	4.5 ± 1.1	[90]
AlZn_5.1_Mg_1.9_Cu_1.47_ Si_2.9_Zr_1.0_	LPBF	As-fabricated	/	446	6.5	[120]
AlZn_7.0_Mg_3.0_Cu_1.0_ Si_2.9_Zr_1.0_	LPBF	As-fabricated	/	421	6.7	[89]
AlMg_4.7_Sc_0.7_Zr_0.3_	LPBF	As-fabricated	/	390 ± 2	24.9 ± 0.6	[91]
		325 °C/4 h	/	531 ± 2	15.0 ± 0.3	
A357 + 0.2Er	LPBF	As-fabricated	/	441.3 ± 6.7	8 ± 1	[103]
AlMn_2.51_Mg_2.7_Sc_0.55_ Cu_0.29_Zn_0.31_	LPBF	As-fabricated	/	350	10	[121]
		325 °C/2 h	/	440	9	

**Figure 8:**
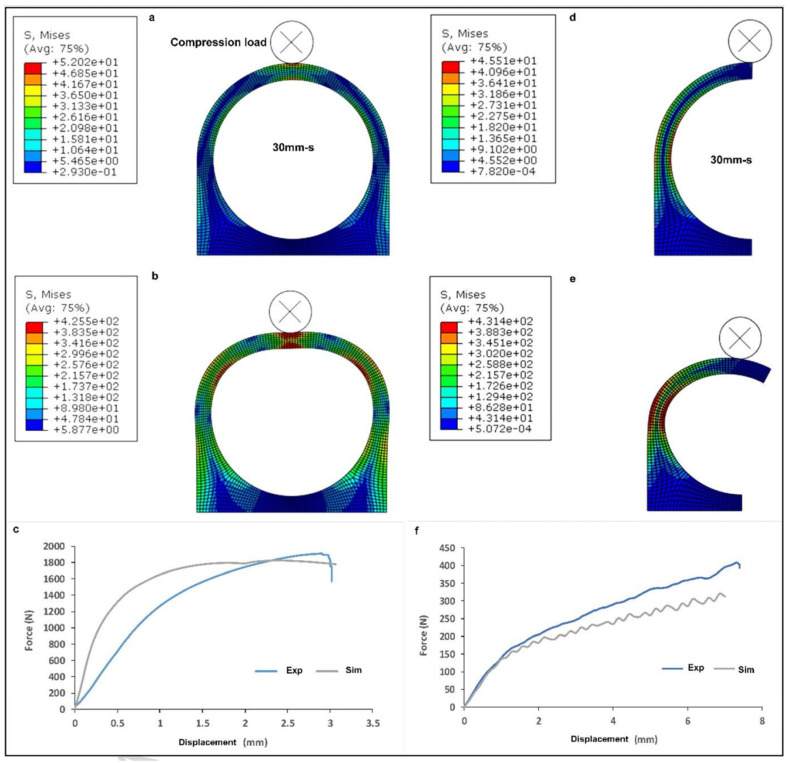
The simulated compressive behavior of 30 mm overhang WPS structures: (**a**–**c**) full-circle overhang; (**d**–**f**) half-circle overhang [122].

Some scholars have conducted numerical simulations to investigate the mechanical properties of LPBF-prepared aluminum alloy parts [122]. The results of these simulations are shown in Figure 8, which demonstrates a close correlation between the maximum compressive strength of the simulation results and that of the experimental results. It is noteworthy to mention that the stiffness of LPBF-prepared aluminum alloy parts is inferior to that of the numerical simulation. This is primarily due to the fact that the LPBF process is employed in the fabrication of overhanging structural components, which results in a reduction in the quality of the parts.

### 3.3. Cost of Aluminum Alloy LPBF Processes

Aluminum alloy has the advantages of lightweight, high specific strength, and good processing performance, so it has been widely used in fields such as aviation and aerospace. Compared with titanium alloys and high-temperature alloys, aluminum alloys are more affordable. The traditional manufacturing processes of aluminum alloys are mature. Hence, the advantage of aluminum alloy LPBF technology in forming complex shapes cannot fully support rapid promotion and application. Cost is also one of the important factors in assessing the application prospects of aluminum-alloy-formed parts. Therefore, the evaluation of the cost of aluminum alloy LPBF technology is crucial. Generally speaking, the main factors that affect LPBF cost include the following five aspects: equipment, raw materials, energy consumption, post-processing, and forming efficiency.

1. Equipment

Equipment is typically the main cost input in the material preparation process. According to the quotes provided by mainstream LPBF equipment vendors on the market, the price of LPBF equipment ranges from CNY 1 million to 4 million (USD 0.14 million to 0.55 million). Large equipment customized for special-shaped formed parts requires over CNY 10 million (USD 1.38 million), mainly because LPBF equipment requires the use of expensive devices such as lasers, powder spreaders, powder sieves, and material dispensers. In addition to the purchase cost of equipment, the cost of use and maintenance must also be considered when calculating the costs. For example, the use of consumables such as filter cotton, filter screens, and scrapers will increase the cost of LPBF processes [123]. The maintenance and replacement of components such as lasers and galvanometers will also lead to increased maintenance costs later on.

2. Raw materials

The LPBF process uses powder materials as raw materials. The composition uniformity, particle size distribution, flowability, and loose packing density of the powder are important factors that affect the quality of LPBF parts [124]. High-quality powder has always been expensive, which is different from the wire and ingot materials used in traditional processes. Compared with wire and ingot materials, the cost of aluminum alloy powder has remained at a higher level in the LPBF process, as shown in Table 3. In addition, the LPBF process requires the use of argon for protection. However, due to the easy oxidation of aluminum alloy, the oxygen content must be kept at a low level, which will increase the use of argon.

Furthermore, there are a significant number of waste aluminum alloy products in cities. By sorting and recycling these aluminum products, the cost of aluminum alloy powder could be further reduced. However, no published reports of related studies have been found.

3. Energy consumption

In recent years, with the increasing demands for “carbon peaking” and “carbon neutrality”, green and low-carbon have become an important requirement for the development of the manufacturing industry. High energy consumption will inevitably lead to a significant increase in costs. Generally speaking, the energy consumption of the LPBF process using laser as the heat source is relatively low when the laser power is low. For example, the rated power of EOS Company’s LPBF printer is approximately 2.4 kW [125]. When calculating energy consumption, not only the power of the equipment should be considered, but also the efficiency of forming should be comprehensively considered. After calculation, the energy consumption of a typical LPBF process is about 75 W·h/cm^3^.

4. Post-processing

Although the LPBF process can achieve near-net shaping, it is limited in terms of key surface roughness, residual stress, and fatigue performance of the formed parts, and therefore, post-processing is usually required [67,109,126]. Common post-processing procedures include heat treatment, support removal, powder cleaning, and secondary processing of the formed parts.

The LPBF process has the characteristic of rapid heating and cooling [127], which results in significant residual stress inside the formed part. If stress relief treatment is not carried out, deformation and fracture are likely to occur in the formed part. Heat treatment is a common method for stress relief, although the performance of the formed part may be reduced after heat treatment. However, for the LPBF process, this process is essential. In addition, when the thickness of the formed part is large, uneven heat treatment may occur, which may affect the pass rate of the formed part. Furthermore, the most complex-shaped parts printed by LPBF still require support. For complex structures with thin walls or internal microchannels, the time for support removal and powder cleaning may even exceed the time for LPBF shaping.

5. Forming efficiency

**Figure 9:**
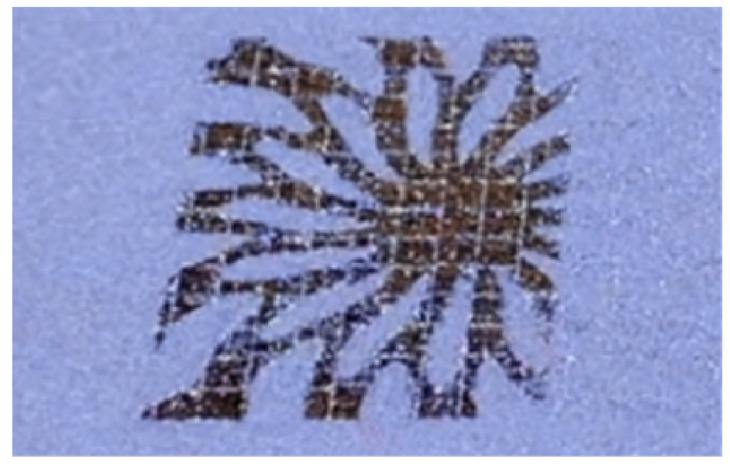
Illustration of AP process [131].

Forming efficiency is directly related to the working hours required for production, and therefore directly affects the manufacturing cost of the formed parts. The higher the efficiency, the lower the manufacturing cost of the formed parts. Generally, the forming efficiency of LPBF is mainly affected by the state of the heat source. Table 4 shows typical technical parameters for the LPBF process. Since the LPBF process uses a small laser spot size for forming, its printing accuracy is relatively high but its forming efficiency is relatively limited. The forming efficiency of LPBF equipment using a single laser is approximately 20 cm^3^/h [128]. Multi-laser systems are one effective way to improve the forming efficiency of LPBF. For example, SLM Solutions has introduced the NXG XII 600, a 12-laser LPBF printer that has been put into mass production, and its forming efficiency has been increased by more than 20 times [129,130]. Of course, the coordination control between multiple laser heads and complex thermal field issues also limit the further increase in the number of printing heads. Seurat Technologies has developed an “Area Printing” (AP) process based on the LPBF process, as shown in Figure 9. By controlling the scanning of 2 million laser points, this process achieves a significantly higher printing efficiency (>1000 cm^3^/h) [131] compared to traditional LPBF processes.

**Figure 10:**
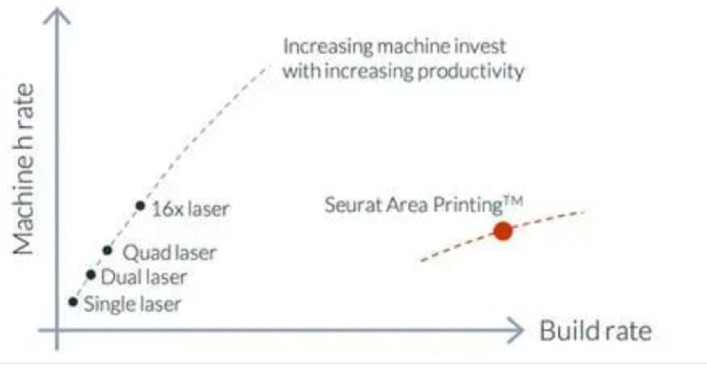
Deposition rate of AP process [131].

Therefore, in summary, the manufacturing cost of aluminum alloy formed by the LPBF process is much higher than traditional machining methods. Equipment procurement and maintenance costs, as well as raw material costs, are the main influencing factors. Although manufacturing cost has not yet become the main factor affecting the application of LPBF aluminum alloy formed parts, as LPBF technology continues to be developed and popularized in civilian fields, the manufacturing cost will gradually become a bottleneck restricting the development of aluminum alloy LPBF (Figure 10).

In this study, two representative aluminum alloy parts were selected to assess the manufacturing cost of traditional manufacturing technology compared with that of the LPBF process. The retail prices of aluminum alloy profiles and aluminum alloy bicycle frames are 22 CNY/kg (3.04 USD/kg) and 1350 CNY/kg (186.3 USD/kg), respectively. A gross profit margin of 20% is applied to the traditional manufacturing costs of aluminum alloy profiles and aluminum alloy bicycle frames, resulting in a cost of 17.6 CNY/kg (2.43 USD/kg) and 1080 CNY/kg (149.04 USD/kg), respectively. The quotation provided by the LPBF supplier in China indicated that the price of printing AlSi10Mg parts was approximately 3500 CNY/kg (483 USD/kg), while the manufacturing cost of LPBF made of aluminum alloy was approximately 2100 CNY/kg (289.8 USD/kg), based on a gross profit margin of 40%. It can be observed that the current LPBF manufacturing cost for aluminum alloys is considerably higher than that of traditional manufacturing methods. The former is 2–120 times higher than the latter, with the discrepancy depending on the complexity of the part. Consequently, the LPBF process is more suitable for the manufacture of highly complex, high-value-added aluminum alloy parts, such as hinges and watch cases.

### 3.4. Applications

Aluminum alloys have many advantages, such as high specific strength, high specific modulus, and high reflectivity, which make them suitable for use as both lightweight high-strength load-bearing structures and functional non-load-bearing structures.

**Figure 11:**
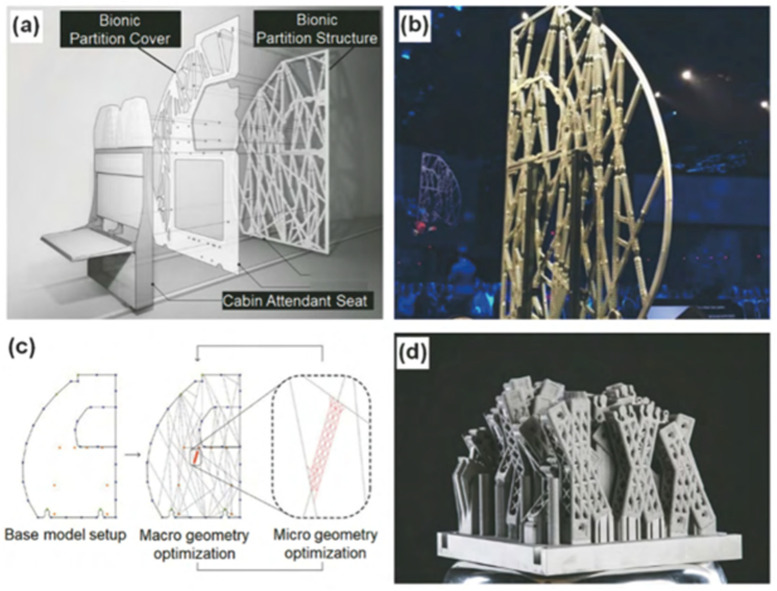
Cabin partition printed by LPBF process (Airbus Co.) [72]. (**a**) Structure of bionic lattice cabin partition; (**b**) photograph of assembled bionic lattice cabin partition; (**c**) cross-scale structure design of bionic lattice cabin partition; (**d**) components of bionic partition processed by SLM additive manufacturing.

With the increase in the variety of aluminum alloy LPBF materials and the progress of printing technology, LPBF aluminum alloy load-bearing structure parts have gradually been put into engineering applications. In 2015, Airbus began using LPBF technology to produce aviation-grade aluminum alloy structural supports for satellite telemetry and remote control antennas. The support replaces four independent components, reducing weight by 35% and increasing hardness by 40% [132]. Based on biomimetic lattice structure design, Airbus has also designed and manufactured commercial aircraft cabin partitions, as shown in Figure 11. Compared with the original honeycomb composite partition, the biomimetic lattice partition manufactured using LPBF technology has reduced the impact displacement by 8%, reduced weight by 45%, and reduced carbon emissions by 465,000 tons per year [72], achieving both economic and environmental benefits. In addition, complex-shaped aluminum alloy structural parts manufactured using LPBF technology have also been applied in the Chang’e-4 relay satellite [133].

**Figure 12:**
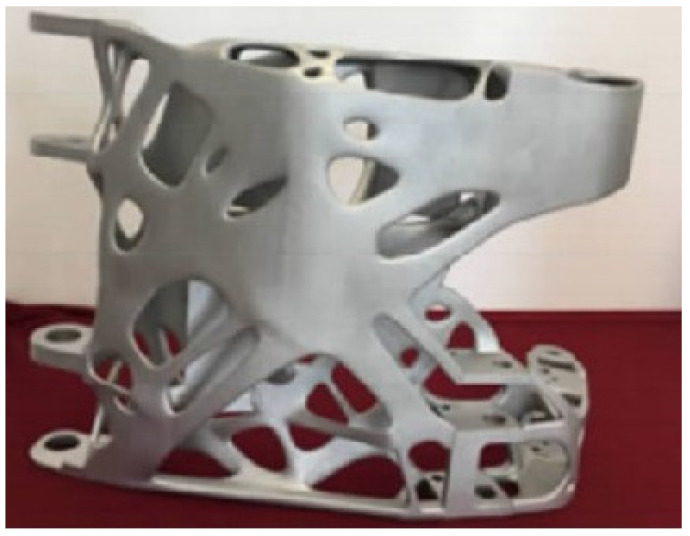
Hinge arm printed by LPBF process [27].

In 2016, Thales Alenia Space teamed up with French company Poly-Shape SAS to provide additively manufactured antenna brackets for South Korea’s new communication satellites, Koreasat-5A and Koreasat-7. The AISi7Mg alloy antenna bracket structure adopts a biomimetic design, combining nine formed parts into one formed part with a final weight of only 1.13 kg, a 22% reduction in weight, and a 30% reduction in cost [134]. The Beijing Aircraft Technology Research Institute of Commercial Aircraft Corporation of China Ltd. (Beijing, China) optimized the structure of the hinge-arm-formed parts of aircraft cabin doors and used LPBF technology for manufacturing (Figure 12). This greatly shortened the production cycle and reduced the weight by 35% [27].

In 2019, AmPro-Innovations Co., Ltd. (Notting Hill, Australia), used a high-strength and tough AM-specific aluminum alloy, AL250C, to manufacture the world’s first full-size LPBF aircraft engine. The yield strength can reach 580 MPa, the tensile strength exceeds 590 MPa, and the elongation is 11%. The engine has a service life of more than 5000 h at 250 °C. The development cycle of the aircraft engine was shortened by 75% compared to traditional development methods, and the delivery time was reduced by 50% [135]. In 2023, Bright Laser Technologies Co., Ltd. (Xi’an, China), selected AlSi10Mg aluminum alloy using LPBF technology to manufacture satellite deployer main frames and cabin doors for the “Dalian No. 1-Lianli” satellite. The main frame structure has a size of about 400 mm × 400 mm × 500 mm with a minimum wall thickness of 1 mm [136].

BMW also applied LPBF technology to the integrated manufacturing of high-precision aluminum alloy water pump wheels for racing cars, replacing the original combined plastic water pump wheels. About 500 LPBF water pump wheels were assembled, achieving small-batch production [137]. Domestic manufacturers have also begun to explore the use of LPBF technology to manufacture automotive-formed parts. Compared with traditional cast aluminum alloy hubs, the lattice-shaped aluminum alloy hub manufactured using LPBF technology has reduced weight by 13%.

Optical systems are important tools for information collection in spacecraft, and metal reflective mirrors are indispensable and widely used in large space telescopes, nano-satellites, and laser radar systems as a typical non-load-bearing structure. LPBF aluminum alloy mirrors have advantages such as a short manufacturing cycle, good processing performance, and easy realization of structural optimization design, with related research starting earlier in foreign countries.

**Figure 13:**
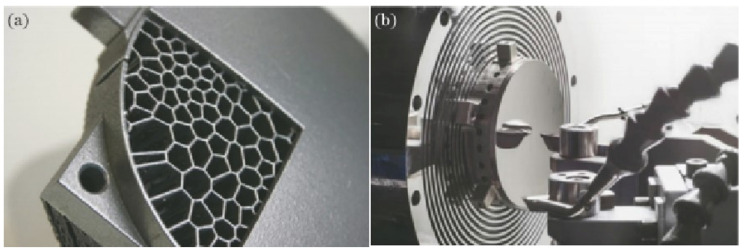
Lightweight metal mirror manufactured by Fraunhofer Institute in Germany using LPBF tech [142]. (**a**) Hollow structure inside the mirror; (**b**) physical diagram of the mirror.

In 2015, Corning Incorporated used laser LPBF technology to manufacture high-performance aluminum alloy mirrors, whose structure is similar to that of traditional mirrors but with higher efficiency in processing and forming [138,139]. Additionally, secondary design optimization through lightweight and topology optimization, followed by LPBF manufacturing, can further improve the performance of the mirrors, as shown in [140,141]. In 2018, the Fraunhofer Institute in Germany used AlSi40 alloy as raw material and LPBF technology to manufacture ultra-lightweight metal reflective mirrors with hollow internal structures, as shown in Figure 13. The hollow structure can greatly improve the weight reduction effect and stability of the mirrors [142].

There is relatively little research on LPBF aluminum alloy mirrors in China. Shanghai Micro Electronics Equipment (Group) Co., Ltd. (Shanghai, China) used laser LPBF technology to try out lightweight, high-rigidity, and high-modal aluminum alloy mirrors. The mirror structure was redesigned through topology optimization, followed by LPBF manufacturing. Compared to the traditional design and manufacturing scheme, the weight of the LPBF reflective aluminum mirror was reduced by 19.5%, the maximum deformation was reduced by 34.9%, and the first-order mode was improved by 36.1% [143].

In conclusion, the current aluminum alloy LPBF technology mainly combines the low-density and high-strength characteristics of aluminum alloy with the advantages of LPBF complex structure forming to achieve structural weight reduction and integrated forming, indirectly producing economic benefits. Its applications are mainly concentrated in low-cost sensitivity and high-value-added fields such as aviation and aerospace. Aluminum alloy LPBF technology is less applied in civilian fields with high-cost sensitivity and low structural complexity, such as automobiles, machinery, and building materials. Compared with traditional manufacturing methods, its manufacturing cost is relatively high, and its advantages are difficult to fully utilize.

## 4. Summary and Future Development Trends

### 4.1. Developing Aluminum Matrix Composite Materials Reinforced with Nanoceramic Particles

There are many types of traditional aluminum alloys with excellent mechanical properties. However, due to the low laser absorption rate, high thermal conductivity of aluminum alloys, and rapid thermal cycling during the LPBF process, most traditional aluminum alloy grades cannot be directly used for LPBF technology. In particular, for the 2XXX and 7XXX high-strength aluminum alloys, their solidification range is large, which makes it easier to produce a large number of pores and microcracks. Currently, the mature and commercially available LPBF aluminum alloys are mainly AlSi alloys, which have low strength, elongation, and modulus and cannot meet the high-performance application requirements in the aerospace industry. After Martin’s research, the AlCuMg, AlMgSi, and AlZnMgCu alloy systems were developed, and the transformation from columnar grains to equiaxed grains was achieved by doping with nanoparticles, which inhibited the formation of hot cracks and successfully achieved the dense formation of 7075 alloys, providing research inspiration for more researchers [88].

In addition, to address the low strength and ductility characteristics of AlSi alloys, particle-reinforced AlSi10Mg composite materials have been developed, such as graphene, SiC, TiO2, TiC, AlN, and TiCN ceramic particles [144,145,146]. Studies have shown that with the reinforcement of nanoceramic particles, only a low content (2%) is required to achieve strength and toughness comparable to high-strength aluminum alloys (>500 MPa). However, nanoparticles tend to aggregate, and achieving uniform dispersion of high-quality fraction ceramic particles remains a major problem to be solved. By using micro-ceramic particle reinforcement, a higher content (>10%) of ceramic particles can be added, but the mismatch of the strength and toughness of the aluminum matrix composite material is a problem that needs to be solved. Based on the principle of grain refinement, the mixed addition of micro and nanoparticles is a feasible way to achieve a breakthrough in the strength and toughness of high-quality fraction aluminum matrix composite materials.

### 4.2. Developing Micro-Alloyed Aluminum Alloy Powders Specifically Designed for LPBF

In addition to adding ceramic and nano-reinforcing phases, designing alloy compositions for LPBF’s fast heating/cooling, complex thermal cycling, and thermal accumulation characteristics and developing LPBF-specific high-performance aluminum alloys are important ways to solve the problem of the few types and low performance of LPBF aluminum alloys, such as AlSi10Mg (US: 323 MPa, η: 15.3%) [115]. Currently, the development of aluminum alloy microalloying and high-throughput element design methods provides important support for the development of LPBF-specific aluminum alloys, especially in microalloying technology, which has made some breakthroughs. The AlMgScZr high-strength aluminum alloy developed by Airbus has shown enormous potential in strength (US: 531 ± 2 MPa) and elongation (15.0 ± 0.3%) [101], attracting many scholars’ exploration and research. In addition, domestic scholars have successfully prepared LPBF-specific AlMgErZr alloy (US > 500 MPa; η > 15%) [102,103] by replacing expensive Sc with cheaper Er, obtaining excellent mechanical properties.

Currently, the research on micro-alloyed aluminum alloy powders for LPBF is just at the starting stage, with fewer types of aluminum alloys available and a lack of related research on the fatigue and corrosion resistance for aluminum alloys in service. Therefore, further breakthroughs in material types and in-depth research on the service performance of materials will become the key to promoting and applying LPBF-specific aluminum alloys in the aviation and aerospace fields.

### 4.3. Developing New Technology and Equipment to Reduce the Costs of LPBF

Currently, the manufacturing cost of aluminum alloy LPBF processes is much higher than the manufacturing cost of traditional processing methods (almost 2–120 times), which will seriously limit the promotion and application of aluminum alloy LPBF processes in cost-sensitive areas such as automobiles, electronics, and construction, where traditional processing methods are mature. Therefore, in the development of aluminum alloy LPBF processes, breakthroughs must be made in cost control. Generally speaking, equipment and raw materials costs are the two main factors affecting the manufacturing cost of aluminum alloy LPBF. Accelerating the localization and mass production of key components such as laser and galvanometer systems, and promoting the research and development of small-scale, desktop LPBF equipment can reduce the manufacturing and maintenance costs.

In addition, the price of existing aluminum alloy LPBF powders is 10–30 times the price of ingots, further expanding the production capacity of aluminum alloy powders, establishing a short preparation process suitable for aluminum alloy powders from the metallurgical stage, establishing a reasonable powder quality grading system, improving the upstream and downstream industrial chain structure, and gradually reducing the manufacturing cost of aluminum alloy powders is also an important measure. In addition, the development of aluminum alloy recovery, classification, metallurgy, and re-powdering technologies and equipment, the use of existing urban mineral resources, and the reduction of manufacturing costs of aluminum alloy powders to meet the needs of the automotive and construction industries are also important driving forces for the future development of aluminum alloy LPBF technology.

## Figures and Tables

**Table 1:** Cost of aluminum alloy manufactured by traditional process.

Materials	Type	Price CNY (USD)/kg
2014	Bar, Panel	29–42 (4.00–5.80)
2219	Bar, Panel, Pipe	24–26.9 (3.31–3.71)
2A12	Bar, Panel, Pipe	16–32 (2.21–4.42)
5052	Bar, Panel, Pipe	15.8–23 (2.18–3.17)
5005	Bar, Panel, Pipe	17–25 (2.35–3.45)
5A06	Bar, Panel, Pipe	20–32 (2.76–4.42)
6061	Wire, Bar, Panel, Pipe	18–27 (2.48–3.73)
6063	Bar, Panel, Pipe	18–35 (2.48–4.83)
7A04	Wire, Bar, Panel, Pipe	19–26 (2.62–3.59)
7055	Panel, Pipe	42–51 (5.80–7.04)
7075	Bar, Panel, Pipe	26–45 (3.59–6.21)
ZLD201A	Ignot	17–26 (2.35–3.59)
ZL101	Ignot	17–19 (2.35–2.62)
ADC6	Ignot	23.5 (3.24)

**Table 3:** Comparison of the price of aluminum alloy.

Materials	AlSi10Mg		6061		7075	
Type	Powder	Ignot	Powder	Ignot	Powder	Ignot
Price/CNY (USD)	100–200 (13.80–27.60)	20–30 (2.76–4.14)	700–800 (96.61–110.41)	19–23 (2.62–3.17)	400–450 (55.21–62.11)	25–35 (3.45–4.83)

**Table 4:** Typical technical parameters of LPBF.

Process	Powder Diameter μm	Laser Power W	Scanning Speed mm/s	Spot Size mm	Manufacturing Size mm	Ref.
LPBF	10~60	50~1000	50~1000	0.05~0.1	750 × 750 × 500	[54,56]

## Data Availability

Data will be made available on request.
